# Safe and favorable prognosis of thoracic endovascular aortic repair for the low-risk patients with non-acute type B aortic dissection

**DOI:** 10.3389/fcvm.2024.1442800

**Published:** 2024-10-28

**Authors:** Ken Nakamura, Kimihiro Kobayashi, Shingo Nakai, Ri Sho, Shusuke Arai, Ai Ishizawa, Daisuke Watanabe, Shuto Hirooka, Eiichi Ohba, Masahiro Mizumoto, Yoshinori Kuroda, Cholsu Kim, Hideaki Uchino, Takao Shimanuki, Tetsuro Uchida

**Affiliations:** ^1^ Division of Cardiovascular Surgery, Nihonkai General Hospital, Sakata, Japan; ^2^ Second Department of Surgery, Yamagata University Faculty of Medicine, Yamagata, Japan; ^3^ Department of Public Health, Yamagata University Faculty of Medicine, Yamagata, Japan

**Keywords:** uncomplicated Stanford type B aortic dissection, conservative treatment, preemptive thoracic endovascular aortic repair, aortic aneurysm, aortic remodeling

## Abstract

**Objective:**

Preemptive thoracic endovascular aortic repair (TEVAR) has the potential to improve the prognosis of Stanford type B aortic dissection (TBAD), however it is important to determine whether it could be safely performed as a prophylactic treatment. This study aimed to determine the short- and long-term outcomes of preemptive TEVAR for uncomplicated TBAD with a small aortic aneurysm.

**Design:**

Retrospective multicenter analysis.

**Methods:**

We analyzed 212 patients with medically treated uncomplicated subacute TBAD between July 2004 and October 2019 in two Japanese academic centers. The short- and long-term prognosis of patients who underwent preemptive TEVAR and the changes in aortic diameter over time after TEVAR were analyzed. Aorta-related complications, aortic-related death and postoperative complications were recorded and analyzed. Analysis was performed on an intension-to-treat basis.

**Results:**

During follow-up, patients were divided into two groups: optimal medical treatment [OMT; *n* = 185 (87%)] and preemptive TEVAR [*n* = 27 (13%)]. In all cases, aortic enlargement was the reason for therapeutic intervention in the preemptive TEVAR group. Propensity score matching yielded a cohort of 27 control patients with OMT (group A) and 27 patients who underwent preemptive TEVAR (group B). Preoperative characteristics were similar between groups. In group B, only one patient developed type A dissection at a late stage and died from aortic rupture. Freedom from aortic-related death at 1/5/10 years was 100%/92%/92% in group B. Overall growth (mm/year) of max aorta was significantly smaller in the TEVAR group than in the control group (−3.7 ± 2.9 vs. 0.4 ± 5.6, *p* < 0.01), and the diameter of the false lumen was reduced (−8 ± 4.8 vs. −1.3 ± 8.0, *p* < 0.001).

**Conclusions:**

Short- and long-term outcomes of TEVAR for uncomplicated TBAD with a small aortic aneurysm were excellent, with few postoperative complications. After TEVAR, aortic remodeling was observed in the short term, suggesting that it may contribute to the prevention of aortic-related death due to rupture.

## Introduction

Acute aortic dissection, the most common catastrophic aortic event affects per 100,000 people is around 2.9%–4.4% (1, 2), the number of cases reported from Japan ranges from 3 to 10% (3, 4). Although the traditional treatment methods for complicated acute Stanford type B aortic dissection such as impending rupture or a malperfusion syndrome have been surgical treatment, open graft replacement of the proximal descending thoracic aorta. In-hospital mortality is reported to be 29% when open surgery is performed in these patients with complicated acute TBAD (5, 6), and the outcome of treatment for acute complications still needs to be improved. On the other hand, the treatment of acute uncomplicated TBAD has historically been well established (7–9), and recent reports indicate that optimal medical treatment (OMT) is a treatment with good survival rates, including in the later phase (10). However, even with good survival rates in the remote stage, aortic complications such as developing aneurysms and rupture are not infrequent (11), and the pros and cons of early intervention are still being controversial (12). Subacute or chronic aneurysm enlargement in patients with uncomplicated TBAD has been reported to be treated with TEVAR with better outcomes and survival rates (13), however which patients are particularly well treated is not clear. We aggressively perform preemptive TEVAR in patients with uncomplicated TBAD who do not have large aortic diameters but have a tendency toward aneurysmal enlargement. The occurrence of acute complications and remote prognosis of these patients are unknown. In this study, we utilized the resources of two institutions to analyze the short-term and long-term outcomes of patients with uncomplicated TBAD treated with preemptive TEVAR compared with those undergoing OMT.

## Methods

Approval from the Yamagata University Hospital Ethical Committee and patient written consent were obtained (Institutional Review Board #2018-245). All patients were informed about the use of their data for clinical research.

**Figure 1:**
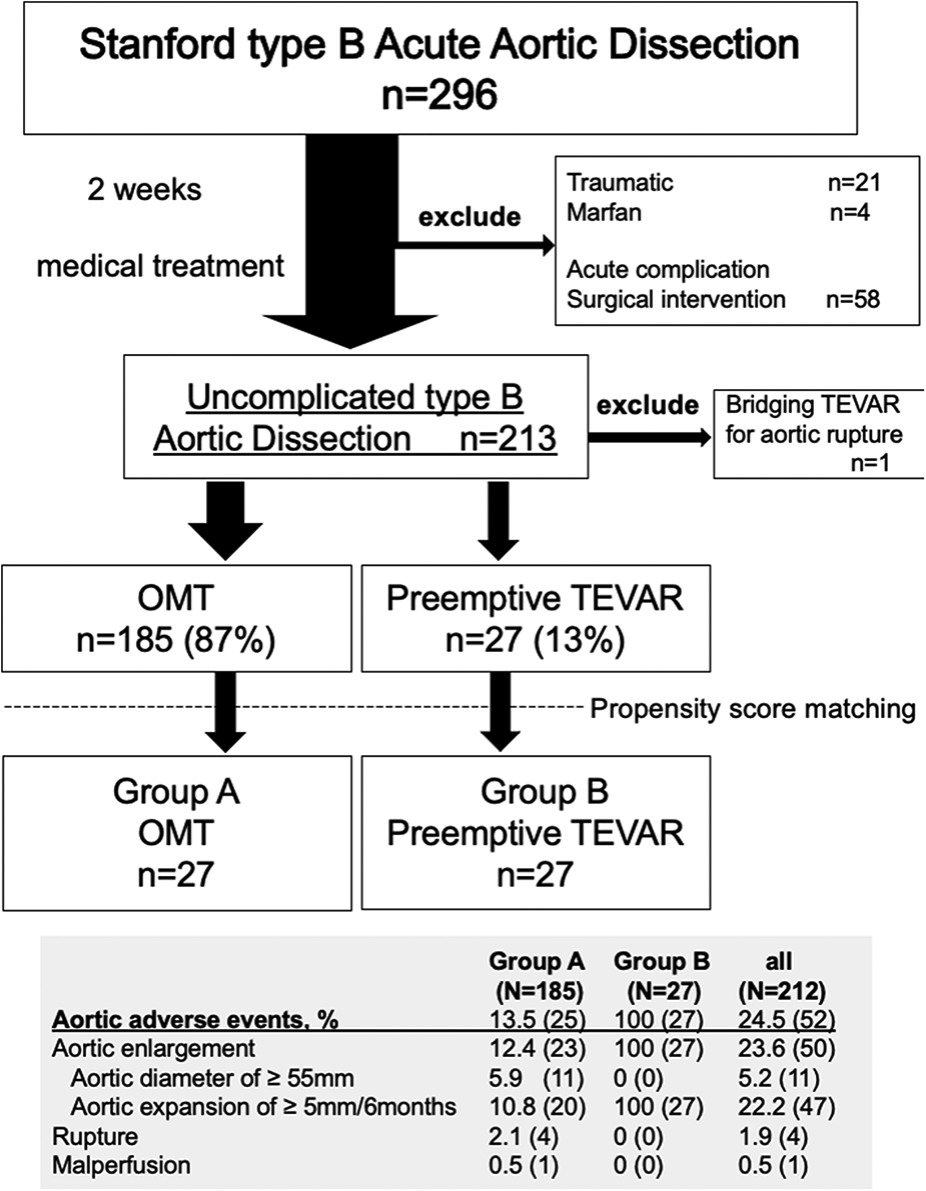
Flow diagram of the entire series of patients with Stanford type B aortic dissection. The study comprised 212 consecutive patients with uncomplicated type B aortic dissection. Propensity score matching was performed and adjusted for the background characteristics of each patient group. Patients were divided into 2 groups according to the treatment option, Group A consisted of 27 patients who received optimal medical treatment (OMT), and Group B consisted of 27 patients who underwent preemptive TEVAR.

Two centers in Yamagata prefecture participated in this study. Data were collected between July 2004 and October 2019 in Yamagata University Hospital and between February 2016 and May 2019 in Nihonkai General Hospital (Figure 1). Acute aortic dissection was defined as a case in which the patient was examined and diagnosed with pain and other symptoms as the main complaints. Acute uncomplicated TBAD was defined as the absence of malperfusion (both dynamic obstruction, which is improved by false lumen decompression, and static obstruction, which is not improved) or signs of early disease progression (14) such as type A dissection presenting within 14 days of symptom onset. We divided the time course of aortic dissection into acute (<14 days), sub-acute (15–90 days), and chronic (>90 days) phases (14). For blood pressure control, all patients received an intravenous calcium-channel blocker, nitroglycerine, *β*-blockers or a combination of these after admission. The systolic blood pressure was controlled to less than 120 mmHg and the heart rate to less than 60, with careful monitoring of urine output starting 2 weeks after onset. Two weeks later, blood pressure was controlled to less than 130 mmHg and heart rate was in the range of 60 to 80. On admission, patients were treated in the intensive care unit (ICU) or a high care unit (HCU). All patients underwent contrast computed tomography (CT) scanning at emergency admission, and on the 1st and 7th days after admission. All patients were administered oral medications starting on the 1st day after CT screening, and were encouraged to take a short walk starting on the 7th day after onset. Patients were eligible for discharge 4 weeks after onset. That protocol was developed using Japanese guidelines as a reference (3). During follow-up, patients who had an aortic adverse event, despite medical management, underwent an aortic intervention. The term “aortic adverse event” includes enlargement of the aortic diameter (≥ 55 mm enlargement and/or ≥ 5 mm enlargement in 6 months), malperfusion and aortic rupture (3, 14). CT angiography (CTA) images at presentation and all follow-up CT scans were reviewed in all patients. The standard scan regimen was as follows: at symptom onset, at discharge, 3 or 6 months after discharge, 12 months after discharge and yearly thereafter. The scan regimen differed for individual patients, depending on findings. All imaging studies were reviewed for radiologic signs of adverse events, such as organ malperfusion or rupture, as well as imaging evidence of pathology resolution. A total of 212 CT images and their analysis were available for routine surveillance. Follow-up CT was absent in 9 patients. Four of them had plain CT, and 13 were excluded from the measurement of cross-sectional area because surgical intervention was performed before the first follow-up (one month after onset).

Image analysis was performed on a SYNAPSE VINCENT system (Tokyo Japan, FUJIFILM Holding Corporation) with a dedicated 3D image analyzer. All CTA measurements were obtained using multiplanar reconstruction (MPR) images in an axial plane perpendicular to the aortic median centerline. The aortic median centerline was generated by software on the VINCENT system that uses a 3D algorithm to perform MPR centered on the contrast-enhanced aortic lumen. In no instance did the maximum aortic diameter occur in a non-dissected segment.

The areas of the true and false lumen were measured. Measurements were performed on the orthogonal cross-section in which the aortic diameter was maximal. Aortic growth rates were calculated by dividing the change in aortic diameter one year after TEVAR by the aortic diameter immediately after TEVAR.

The status of the false lumen on imaging was classified as patent if flow was present in the absence of thrombus. “Partial thrombosis” was evaluated at a later phase when an enhanced CT scan was performed and also classified as “patent”. “Complete thrombosis” was classified as “thrombosed” (15).

Intramural hematomas and penetrating aortic ulcers were not included in this study.

The incidence and percentage for variables of each classification were recorded using descriptive statistics. For continuous variables, we recorded the sample size, mean, standard deviation, and minimum and maximum values. Survival rates were compared using Kaplan– Meier curves and the log-rank test. This study was performed under intention-to-treat analysis. A matched group analysis was performed by propensity matching preemptive TEVAR cases and OMT cases. Propensity scores were generated using logistic regression analyses in 2 steps. Potential predictors were selected from a published data review, known confounding covariates for the outcomes of interest, differences between the 2 patients groups (Table 1), and clinical judgement. The groups were matched with age, sex, height, weight, body mass index, history of chronic obstructive pulmonary disease, hypertension, diabetes mellitus and stroke. Univariate analysis was performed using ANOVA or Student's *t*-test and the Chi-square test. The analysis of aortic diameter with follow-up outcomes was calculated with Repeated Measures Analysis of Variance (RMANOVA). All statistical analyses were performed with JMP software, ver. 17 (SAS Institute, Tokyo, Japan).

**Table 1:** Baseline patient characteristics of all patients with or without an aortic adverse event.

Patient characteristics	All patients (*n* = 212)	OMT (*N* = 27)	Preemptive TEVAR (*N* = 27)	*p* value
Age, y, mean ± SD	70.0 ± 11.9	68.6 ± 15.2	68.0 ± 9.8	0.865
Male, %	70 (148 of 212)	85 (23 of 27)	70 (19 of 27)	0.327
Height, cm, mean ± SD	161.0 ± 9.7	164.2 ± 8.6	161.8 ± 9.4	0.326
Weight, kg, mean ± SD	61.2 ± 14.5	68.2 ± 15.0	66.6 ± 14.5	0.7
BMI (kg/m^2^), mean ± SD	23.4 ± 4.3	25.2 ± 4.6	25.3 ± 4.4	0.914
COPD, %	39 (82 of 210)	37 (10 of 27)	33 (9 of 27)	1
HTN, %	51 (109 of 212)	70 (19 of 27)	70 (19 of 27)	1
Diabetes mellitus, %	6.1 (13 of 212)	11.1 (3 of 27)	14.8 (4 of 27)	1
History of stroke, %	3.8 (8 of 212)	0 (0 of 27)	0 (0 of 27)	–
Renal insufficiency, %	6.6 (14 of 212)	0 (0 of 27)	3.7 (1 of 27)	1
Coronary artery disease, %	3.8 (8 of 212)	3.7 (1 of 27)	7.4 (2 of 27)	1
Hyperlipidemia, %	33 (70 of 212)	55.6 (15 of 27)	44 (12 of 27)	0.587
Follow up period, months, mean ± SD	55 ± 45	47.3 ± 40	51.3 ± 31	0.681
Acute course of disease				
Delirium, %	44 (89 of 204)	26 (7 of 27)	44 (11 of 25)	0.245
NPPV required, %	27 (55 of 204)	30 (8 of 27)	40 (10 of 25)	0.562
Tracheal Intubation, %	10 (20 of 204)	0 (0 of 27)	8 (2 of 25)	0.226
Positive pressure ventilation required (NPPV or mechanical ventilation), %	32 (65 of 204)	22 (6 of 27)	32 (8 of 25)	0.536
ICU + HCU stay, day Mean ± SD	6.2 ± 4.9	5.4 ± 4.6	5.9 ± 3.8	0.687
Medication at discharge from the hospital				
Beta blockers *n*, %	78 (161 of 207)	92 (24 of 26)	82 (22 of 27)	0.25
Angiotensin converting enzyme inhibitors, %	19 (39 of 207)	7.7 (2 of 26)	22 (6 of 27)	0.604
Angiotensin II receptor blockers, %	65 (135 of 207)	69 (18 of 26)	59 (16 of 27)	0.569
Calcium channel blockers, %	77 (160 of 207)	73 (19 of 26)	67 (18 of 27)	0.766
Statins, %	31 (64 of 207)	58 (15 of 26)	40 (11 of 27)	0.276
Steroids, %	4 (8 of 211)	0 (0 of 26)	8 (2 of 26)	0.236
Anticoagulants, %	5 (11 of 212)	0 (0 of 27)	4 (1 of 27)	1

TEVAR, thoracic endovascular aortic repair; OMT, optimal medical treatment; SD, standard deviation; BMI, body mass index; COPD, chronic obstructive pulmonary disease; HTN, hypertension; NPPV, noninvasive positive pressure ventilation; ICU; intensive care unit; HCU, high care unit.

## Results

A total of 296 patients were admitted with a diagnosis of TBAD. A total of 212 medically treated patients were enrolled. All were Japanese, and 148 (70%) were males. The mean (range) age of all patients was 70 (62.0–79.0) years. The most common comorbidity was hypertension (*n* = 109, 51%); diabetes mellitus and renal insufficiency were present in 6.1% (13 of 212) and 6.6% (14 of 212) of the study population, respectively. The average observation period was 55 ± 45 months (Table 1). One of the patients who underwent TEVAR in the subacute phase had TEVAR as a bridging procedure until graft replacement for aortoesophageal fistula and was excluded from the present study.

**Figure 2:**
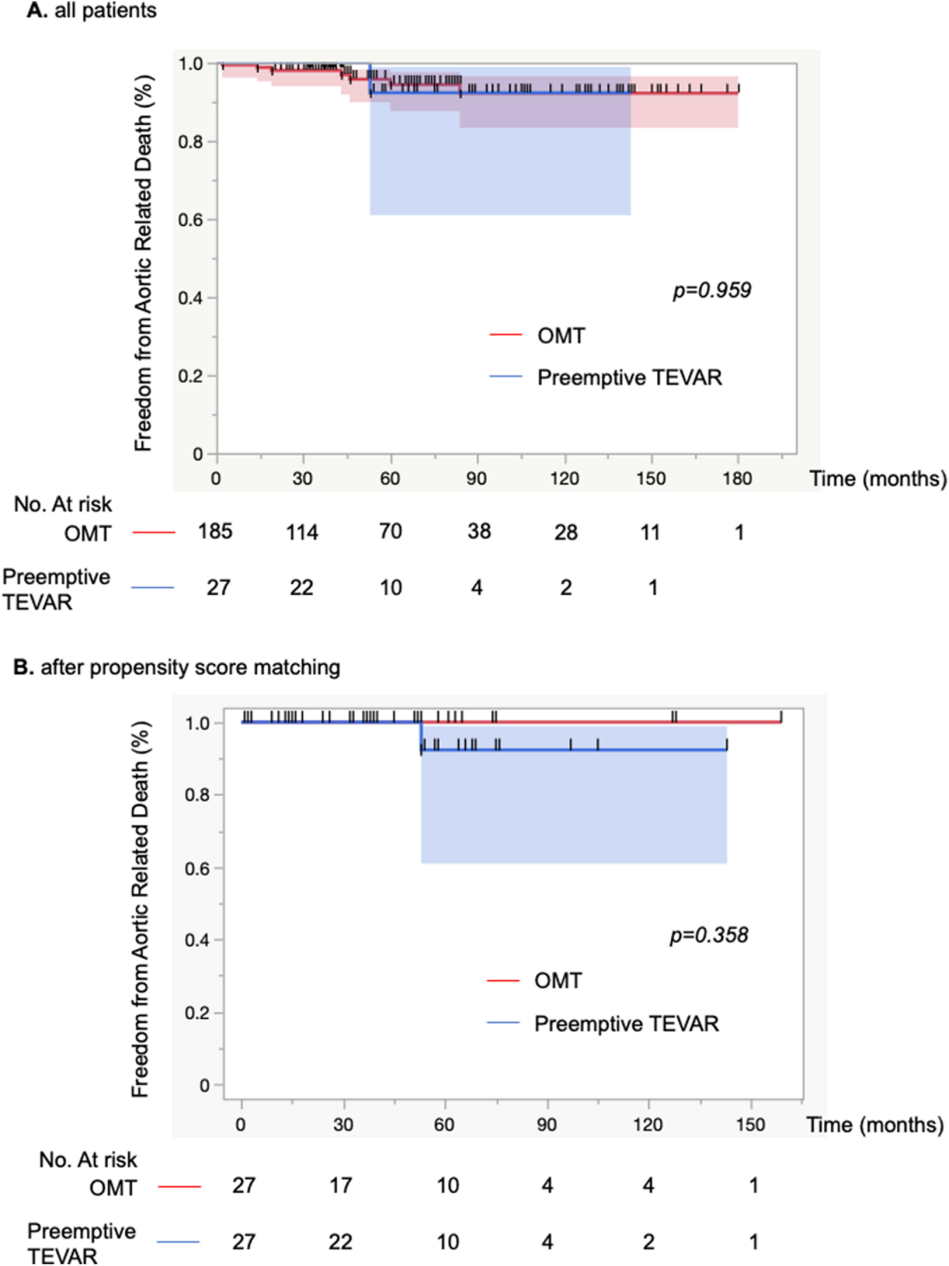
**(A)** Kaplan-Meier curve of freedom from aortic-related death for 212 patients. Optimal medical treatment (OMT) was performed on the basis of conservative treatment, and if aortic adverse events occurred during the course of the disease, graft replacement was performed. **(B)** Kaplan-Meier curve of freedom from aortic-related deaths for 54 patients. Propensity score matching was performed and adjusted for the background characteristics of each patient group. Comparisons between groups were made with the log-rank test.

During follow-up, a total of 27 patients (12.7%; group B) underwent preemptive TEVAR. All of the indications for TEVAR were aortic enlargement, none of which met the definition of maximum aortic diameter of >55 mm due to short-term enlargement. In the OMT group (group A), the incidence of aortic rupture was 1.9% (*n* = 4), and 1 patient (0.5%) required surgical management within 1 month of onset of dissection due to malperfusion (Figure 1). Four patients died from aortic adverse events (1.9%). An 84-year-old man died of aortobronchial fistulation 2 months after onset. He had dementia and could not stand by himself, and his family did not request surgical intervention. Three other patients died of aortic rupture, two of whom died after graft replacement. Aortic interventions were needed in 39 patients (18%), and these included 13 with graft replacement, 27 with endovascular repair, 2 with hybrid (open and endovascular) repair and 1 with an extra-anatomical bypass. Two patients who developed Stanford type A aortic dissection during the follow-up period underwent graft replacement, and both survived. One death occurred from type A dissection 14 months after preemptive TEVAR, but the relationship to TEVAR was unclear. The survival rate after surgical intervention was 92% (35 of 38). The 1-, 5-, and 10-year aortic-related death-free rates for groups A and B were 99%/94%/92% and 100%/92%/92%, respectively (Figure 2A).

**Figure 3:**
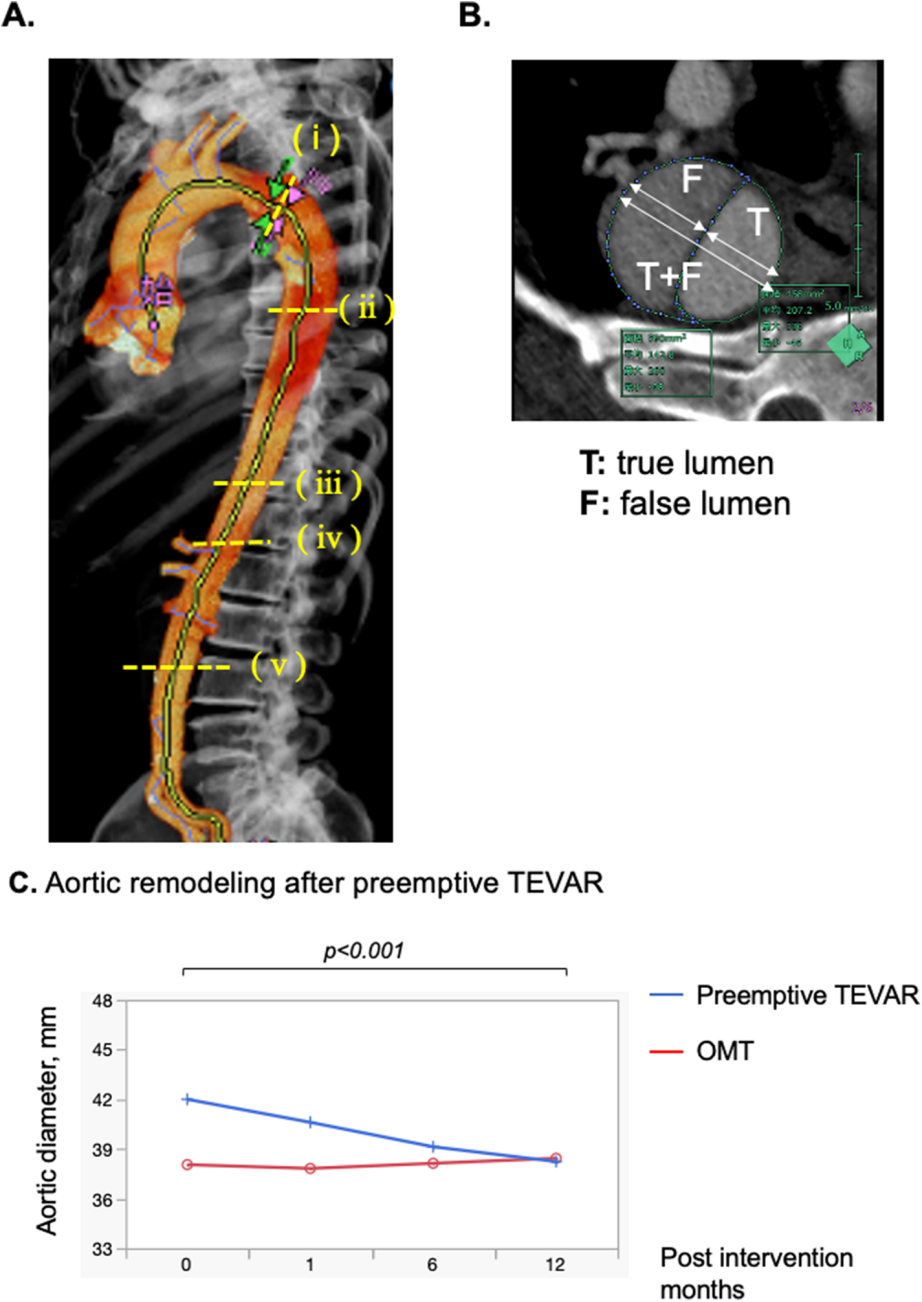
**(A)** Computed tomography (CT) measurement techniques. Aortic dissection was diagnosed via contrast-enhanced CT in all patients. On CT images obtained at admission, the aortic diameter and aortic area were measured, and false lumen patency was assessed. 3D image of the aorta after generation of an aortic centerline from the proximal aortic arch to the left iliac artery by software on the VINCENT system. The aortic diameters and area were measured at 5 locations based on the anatomical site, comprising the distal aortic arch, tracheal bifurcation, diaphragm, celiac artery and abdominal aorta (level of inferior mesenteric artery branch off). **(B)** Measurement of diameter of the true and false lumens and the diameter of the true and false lumens **(C)** maximum aortic diameter (mm) before TEVAR and 1, 3, 6, and 12 months after TEVAR were measured, and measurements were plotted. Compared with Preemptive TEVAR group and optimal medical treatment group. The 95% confidence interval for the mean is shown with the graph.

Demographics, comorbid conditions, and dissection characteristics based on the initial CT findings were compared between groups with propensity matching (Tables 1, 2). There were no differences in patient's background. The Kaplan-Meier curve of freedom from aortic-related death after propensity matching also showed no significant difference between groups (Figure 2B). Marfan syndrome was present in 4 patients overall, and those patients were excluded from the analysis. Acute observations showed no difference between the groups in occurrence of delirium, tracheal intubation, and length of stay in the HCU and ICU (Table 1). At discharge, no differences between groups were detected in medication (Table 1). From the initial CT findings, no significant difference between groups was found in the number of intimal tears (group A: 1.8 ± 1.1 vs. group B: 2.3 ± 1.4, *p* = 0.21), but patent false lumen was more common in group A vs. group B [70% (19 of 27)] vs. 33% [9 of 27], respectively; *p* = 0.014; Table 2). There was no significant difference between groups in initial aortic diameter on admission (group A: 38 ± 6 vs. group B: 38 ± 6, *p* = 0.775), maximum aortic area (group A: 1,251 ± 557 mm^2^ vs. group B: 1,220 ± 363 mm^2^, *p* = 0.814) and the location of entry tears (Table 2) in the late-phase CT findings. The maximum aortic diameter at the initial CT scan of group B was small, with a maximum value of 47 mm and a minimum value of 20.5 mm. Aortic diameter was significantly enlarged in group B (group A: 1.8 ± 5.6 vs. group B: 10.8 ± 8, *p* < 0.0001). Primary entry was most common in the distal arch (78%, 21 of 27), followed by the celiac artery (15%, 4 of 27) and diaphragm levels (7%, 2 of 27). All TEVARs were performed with the objective of closing the primary entry, and 100% were successful. All false lumens at the location of the entry tear were thrombosed after TEVAR, and the average time required for complete thrombosis to form was 1.8 ± 2.1 months. Results of aortic diameter shows overall growth (mm/year) of max aorta was significantly smaller in the TEVAR group than the control group (−3.7 ± 2.9 vs. 0.4 ± 5.6, *p* < 0.01). The monthly post-TEVAR aortic diameter (within and between the group interaction) was significantly reduced after TEVAR (*p* < 0.001) (Figure 3C).

**Table 2:** Initial computed tomography findings for all patients with or without an aortic adverse event.

Initial findings	All patients (*n* = 212)	OMT (*N* = 26)	Preemptive TEVAR (*N* = 27)	*p* value
Aortic diameter on admission, mm, Mean ± SD	38 ± 6	38 ± 6	38 ± 6	0.755
Initial true and false lumen area of maximum aortic diameter (mm^2^)	1,232 ± 495	1,223 ± 603	1,287 ± 350	0.634
True lumen area of maximum aortic diameter (mm^2^)	644 ± 284	640 ± 300	584 ± 253	0.469
False lumen area of maximum aortic diameter (mm^2^)	588 ± 404	629 ± 450	702 ± 275	0.478
True lumen area ratio (true/false + true)		0.58 ± 0.05	0.43 ± 0.19	0.018
Frue lumen area ratio (false/false + true)		0.43 ± 0.23	0.56 ± 0.21	0.034
Entry located distal arch, %	79 (157 of 200)	73 (19 of 26)	78 (21 of 27)	0.757
Entry located tracheal bifurcation level, %	3 (6 of 200)	0 (0 of 26)	0 (0 of 27)	-
Entry located diaphragm level, %	10 (20 of 200)	23 (6 of 26)	7 (2 of 27)	0.142
Entry located celiac artery level, %	6 (12 of 200)	0 (0 of 26)	15 (4 of 27)	0.111
Entry located abdominal aorta, %	3 (6 of 200)	4 (1 of 26)	0 (0 of 27)	0.491
Number of intimal tears	1.7 ± 1.1	1.8 ± 1.1	2.3 ± 1.4	0.21
Diameter of primary entry, mm, Mean ± SD	12.7 ± 7.3	10.8 ± 3.3	12.7 ± 7.0	0.506
Patent false lumen, %	40 (85 of 212)	33 (9 of 27)	70 (19 of 27)	0.014
Follow-up findings				
Aortic growth rate (mm/month)	0.48 ± 1.12	0.3 ± 0.93	1.8 ± 1.33	<0.0001

OMT, optimal medical treatment; TEVAR, thoracic endovascular aortic repair; SD, standard deviation.

Although the diameter of the true lumen was not changed (3.5 ± 0.7 vs. 2.8 ± 7.1, *p* = 0.261), the diameter of the false lumen was reduced (−8 ± 4.8 vs. −1.3 ± 8.0, *p* < 0.001) (Table 3). Remodeling of the stenting site was significantly more pronounced in the first month (true lumen: 2.8 ± 2.7 mm, *p* < 0.0001, false lumen: −4.0 ± 3.9 mm, *p* < 0.0001, true + false lumen: −1.4 ± 2.2 mm, *p* < 0.0001) than in the subsequent six months for true lumen diameter, false lumen diameter and true + false lumen aortic diameter (true lumen: 0.7 ± 2.1 mm, *p* = 0.706, false lumen: −2.0 ± 2.9 mm, *p* = 0.912, true + false: −1.5 ± 2.0 mm, *p* = 0.0295; Supplementary Figure S1). Overall growth (mm/year) and growth rates (%) of distal aortic arch (DAA), level of tracheal bifurcation, diaphragm, celiac artery, and inferior mesenteric artery (IMA) after TEVAR were −4.8 ± 7.7 and (−11.5 ± 19.9), −4.2 ± 7.7 and (−10.7 ± 20.1), −2.6 ± 2.8 and (−6.9 ± 7.7), −1.1 ± 2.7 and (−3.2 ± 8.7), and 0 ± 2.5 and (0.6 ± 9.7), respectively in group A sub analysis (Table 4, Supplementary Figures S2–S6). Growth of the abdominal aorta was minimal. The true lumen tended to enlarge after TEVAR, with the DAA (2.7 ± 7.6 mm/year, 10.6 ± 27.3%) more enlarged and peripherally less enlarged (inferior mesenteric artery level: 1.1 ± 1.9 mm/year, 6.0 ± 11.1%). The diameter of the true lumen of the DAA (*p* = 0.0003) and the level of tracheal bifurcation (*p* = 0.0054) increased significantly during the first year after TEVAR, but no significant difference was observed in the periphery from the diaphragm. In contrast, false lumens tended to shrink after TEVAR, with a greater change in DAA (−7.1 ± 5.3 mm/year, −61.6 ± 35.7%) and less change in peripheral IMA levels (−0.9 ± 3.6 mm/year, 35.1 ± 251.2%) (Supplementary Figures S2–S5). In addition, the diameter of the false lumen of the DAA (*p* < 0.0001), level of tracheal bifurcation (*p* < 0.0001) and diaphragm (*p* < 0.0001) decreased significantly during the first year after TEVAR, but no significant difference was observed in the periphery from the celiac artery.

**Table 3:** Changes in aortic growth and growth rates.

Image findings in 12 months	OMT (*N* = 26)	Preemptive TEVAR (*N* = 27)	*p* value
Aortic diameter before intervention	38 ± 5	43 ± 7	0.0141
Aortic growth (mm/year)	0.4 ± 5.6	−3.7 ± 2.9	0.0019
Aortic growth rates (%)	2.0 ± 12.5	−8.7 ± 6.5	0.0003
True lumen			
Aortic growth (mm/year)	2.8 ± 7.1	3.5 ± 0.7	0.261
Aortic growth rate (%)	15.8 ± 35.2	18.1 ± 15.0	0.763
False lumen			
Aortic growth (mm/year)	−1.3 ± 8.0	−8 ± 4.8	0.0009
Aortic growth rates (%)	−2.9 ± 63.1	−65.7 ± 30.4	<0.0001

OMT, optimal medical treatment; TEVAR, thoracic endovascular aortic repair.

Optimal medical treatment group vs. preemptive thoracic endovascular aortic repair group.

**Table 4:** Changes in aortic growth and growth rates.

Aortic levels	True + False lumen		True lumen		False lumen	
	Aortic growth (mm/year)	Aortic growth rates (%)	Aortic growth (mm/year)	Aortic growth rates (%)	Aortic growth (mm/year)	Aortic growth rates (%)
maximum aorta	−3.6 ± 3.0	−8.7 ± 6.5	4.7 ± 3.5	18.1 ± 15.0	−8.0 ± 4.8	−65.8 ± 30.4
distal aortic arch	−4.8 ± 7.7	−11.5 ± 19.9	2.7 ± 7.6	10.6 ± 27.3	−7.1 ± 5.3	−61.6 ± 35.7
tracheal bifurcation	−4.2 ± 7.7	−10.7 ± 20.1	2.6 ± 8.4	12.6 ± 28.2	−6.4 ± 5.6	−50.2 ± 57.8
diaphragm	−2.6 ± 2.8	−6.9 ± 7.7	1.5 ± 3.7	7.6 ± 15.1	−4.0 ± 3.7	−44.7 ± 35.7
celiac artery	−1.1 ± 2.7	−3.2 ± 8.7	1.0 ± 3.0	6.7 ± 18.6	−1.7 ± 4.9	−22.1 ± 149.7
inferior mesenteric artery	0 ± 2.5	0.6 ± 9.7	1.1 ± 1.9	6.0 ± 11.1	−0.9 ± 3.6	35.1 ± 251.2

TEVAR, thoracic endovascular aortic repair.

Sub analysis of post preemptive thoracic endovascular aortic repair.

## Discussion

In this study, we showed that preemptive TEVAR for uncomplicated TBAD is associated with few postoperative complications, aneurysmal progression, and increased aortic remodeling.

Treatment of uncomplicated TBAD is divided into acute, subacute, and chronic phases depending on the time of disease onset. The 30-day mortality rate for acute type B aortic dissection (AD) is reported to be 10% (16), and malperfusion is responsible for half of this mortality (6). TEVAR in the acute treatment of TBAD is indicated when aortic complications occur, but it is reported that 50% of patients require intervention after 1 year; thus, further improvement is needed. The Investigation of Stent Grafts in Aortic Dissection (INSTEAD) trial comparing TEVAR + best medical treatment (BMT) with BMT alone is well-known for TEVAR outcomes in subacute uncomplicated TBAD, showing the superiority of endovascular aortic repair over BMT alone in terms of survival and remodeling in long-term outcomes, but the number of participants was small (136 patients with chronic dissection), and the use of TEVAR for subacute uncomplicated TBAD has not yet been established (12). A recent meta-analysis confirmed favorable results for uncomplicated and complicated TBAD, showing a significant early mortality advantage of TEVAR over open surgical reconstruction (7.3% vs. 19.0%, respectively; *p* = 0.024) (17). This was due to the lower incidence of paraplegia and stroke with TEVAR (18). Early intervention with TEVAR is a growing indication, but intervention in asymptomatic, uncomplicated patients should be done with caution, and it has not yet been established which patients should be treated. Recently reported predictive factors for aortic enlargement include the number of vessels originating from the false lumen (19) and the number of intercostal arteries (20). Factors that predict aortic events and mortality in patients with chronic TBAD include male sex (21), partial thrombosis of the false lumen (22), aortic diameter >40 mm (7, 23, 24), early aortic expansion after onset (25), younger age (13, 21, 26), a primary entry tear located on the concavity of the distal aortic arch (27) and dissection entry tears >10 mm (25). These risk factors are indications for surgical intervention (28, 29). There are many reports on the aortic diameter at the onset of acute TBAD, and it is an especially important independent risk factor (30); it is highly likely that the diameter of the aneurysm will increase over the long term in patients with an aortic diameter >40 mm at the site of dissection at the initial examination (10). In the case of a small aortic diameter, early complications from preemptive TEVAR are undesirable, but in the present study, patients had good outcomes after TEVAR, and no major complications, such as cerebral infarction or spinal cord paralysis, occurred. The reasons for this may include the fact that we adhered to the TEVAR instructions for use, cases considered technically difficult were not targeted for treatment, and many cases were treated in the subacute phase of the intervention (Table 5). Nienaber et al. reported acceptable early outcomes with preemptive TEVAR, with a mortality of 91.3% and incidence of neurologic adverse events of 4.2% (31). In our study, mortality from preemptive TEVAR within 1 year was 0%, and no adverse events occurred. This is thought to be due to an advantage in patient selection, and demonstrates that preemptive TEVAR can be performed safely when the indications are appropriate. Nienaber et al. reported an average maximum aortic diameter of 44.1 ± 9.6 mm for treated patients, whereas we treated patients with a smaller average diameter of 38 ± 6 mm. In our study, there were no treatment-related complications, indicating that preemptive TEVAR is safe and feasible in patients with early aortic dissection. Aortic remodeling was also mentioned by Nienaber et al., who found that 92.6% had remodeling within 1 year after stenting (31). Our results also showed true lumen expansion and false lumen shrinkage, with remodeling being greater on the proximal side and smaller on the distal side. In our study, early postoperative and long-term outcomes of preemptive TEVAR were favorable, with early aortic remodeling obtained. A particularly important finding of this study was that thrombosis of the largest false lumen of the aorta was obtained in all cases within 1 year after closure of the primary entry with TEVAR. Specifically, 80% (21 of 26) of these cases had thrombosis of the false lumen within 1 month after the procedure. The results of the current study also suggest that performing TEVAR before the dissected aorta undergoes anatomic changes that would result in a massive aneurysm may reduce the risk of mortality and aortic adverse events, such as rupture.

**Table 5:** Timing of therapeutic intervention.

	Preemptive TEVAR (*N* = 28)
Subacute,%	89 (24 of 27)
Chronic,%	11 (3 of 27)

TEVAR, thoracic endovascular aortic repair.

The term “preemptive TEVAR” is used when the strategy is an alternative to prevention and aims to prevent progression of the disease once it has occurred (12, 32). The TEVAR in our study is considered to be included in the preemptive TEVAR, as it is mainly a prophylactic aspect of aneurysm. The definition of “preemptive TEVAR” may require further discussion.

Our study is limited by its retrospective design, relatively small number of patients, incomplete follow-up (9%), and varying number of CT scans among patients. Three cases of Ulcer-like projection type were observed in the present study, all of which became patent false lumen type over time. Therefore, these three cases are classified as patent false lumen in this study. In some cases, surgical intervention was performed when aneurysmal enlargement was observed at a rate equivalent to 5 mm/6 months before 6 months after onset, so we have included 0.83 mm/month as a criterion for aneurysmal enlargement.

These findings highlight the potential of preemptive TEVAR to achieve early favorable outcomes and to prevent future aortic-related events. This work, however, is hypothesis generating and requires prospective validation in larger cohorts. Although our treatment was performed in accordance with the instructions for the use of stent grafts, further studies should be conducted to determine which patients have a better prognosis when preemptive TEVAR is performed.

## Conclusions

Early postoperative complication and survival rates after preemptive TEVAR for subacute or early chronic TBAD were favorable. Rates of future aortic intervention were low, and preemptive TEVAR was performed safely, according to anatomic indications. TEVAR for TBADs with small aortic diameters results in early reduction of the false lumen and is particularly prone to remodeling in the aortic arch and proximal descending aorta. Preemptive TEVAR in appropriate cases may be an progressive option for subacute or chronic TBAD.

## Data Availability

The datasets presented in this study can be found in online repositories. The names of the repository/repositories and accession number(s) can be found in the article/Supplementary Material.

## Supplementary material

Supplemental Figures

Combined diameter of the true and false lumens and the diameter of the true and false lumens before TEVAR and 1, 3, 6, and 12 months after TEVAR were measured, respectively.

Supplementary Figure S1 Maximum aortic diameter.

Supplementary Figure S2 Distal arch of the aorta.

Supplementary Figure S3 Tracheal bifurcation level of the aorta.

Supplementary Figure S4 Diaphragm level of the aorta.

Supplementary Figure S5 Celiac artery level of the aorta.

Supplementary Figure S6 Inferior mesenteric artery level of the aorta.
