# Supplementary figures and images for: Safe and favorable prognosis of thoracic endovascular aortic repair for the low-risk patients with non-acute type B aortic dissection
Source: Front Cardiovasc Med. 2024 Oct 28;11:1442800. doi: 10.3389/fcvm.2024.1442800 (PMC11550929; doi:10.3389/fcvm.2024.1442800)

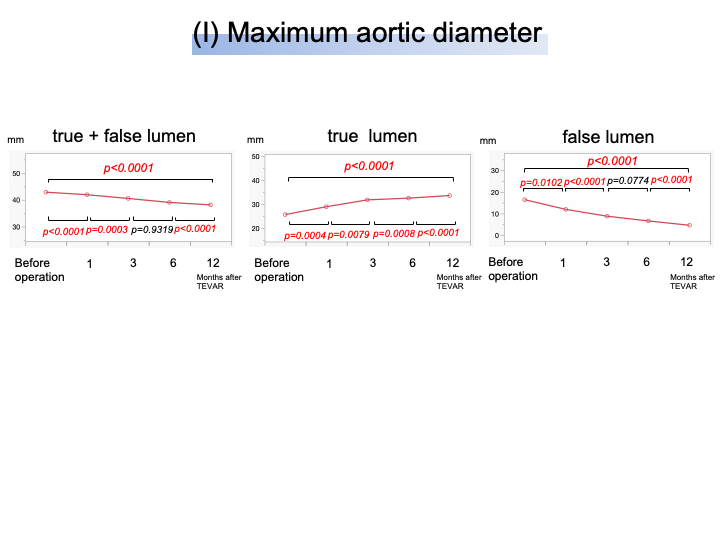

Supplement: Supplementary Figure S1 [file Image1.tiff]

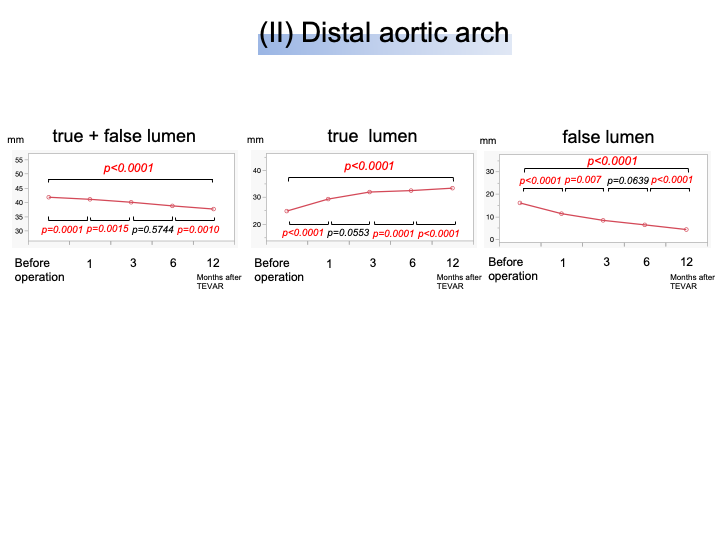

Supplement: Supplementary Figure S2 [file Image2.tiff]

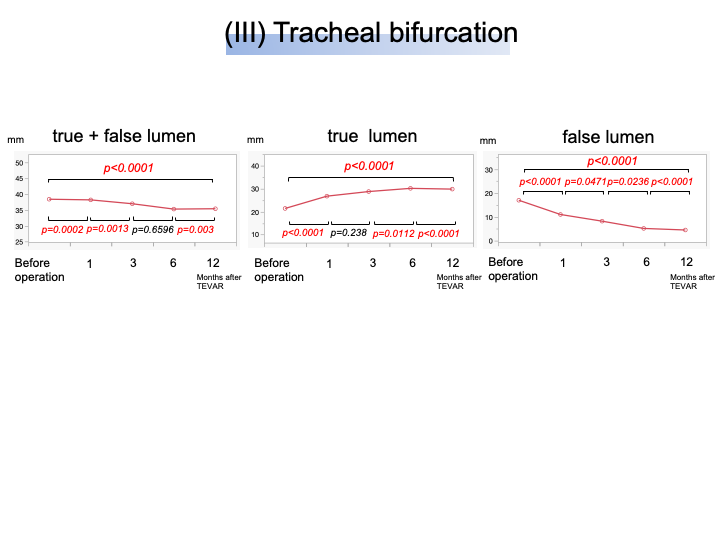

Supplement: Supplementary Figure S3 [file Image3.tiff]

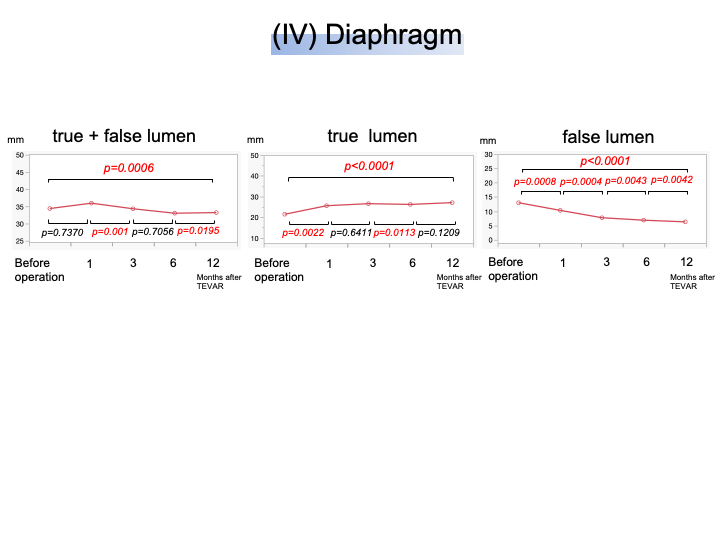

Supplement: Supplementary Figure S4 [file Image4.tiff]

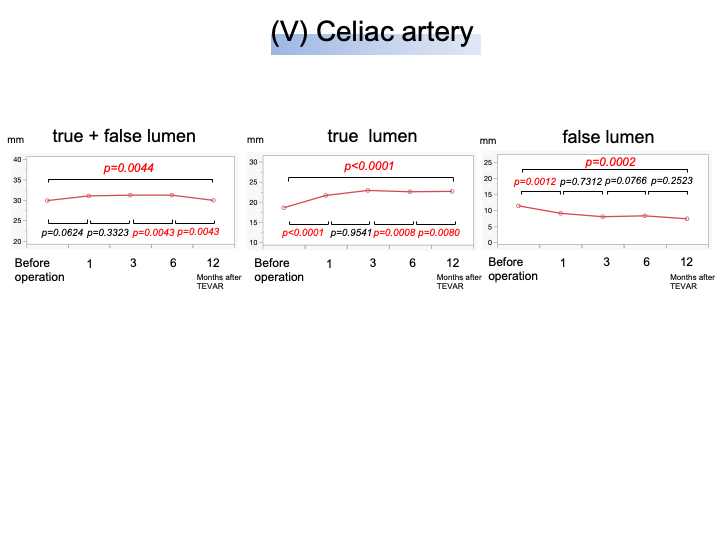

Supplement: Supplementary Figure S5 [file Image5.tiff]

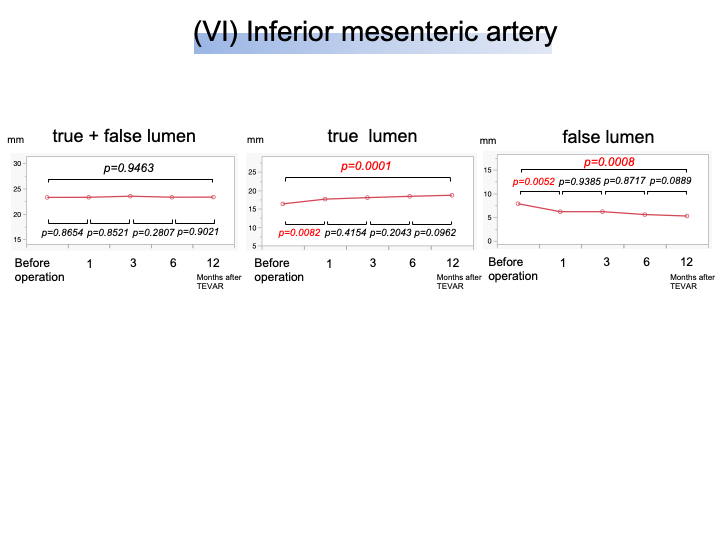

Supplement: Supplementary Figure S6 [file Image6.tiff]
